# Long Term Memory for Noise: Evidence of Robust Encoding of Very Short Temporal Acoustic Patterns

**DOI:** 10.3389/fnins.2016.00490

**Published:** 2016-11-24

**Authors:** Jayalakshmi Viswanathan, Florence Rémy, Nadège Bacon-Macé, Simon J. Thorpe

**Affiliations:** ^1^Centre de Recherche Cerveau et Cognition, Centre National de la Recherche Scientifique UMR 5549Toulouse, France; ^2^Faculty of Medicine, Purpan, University of Toulouse III Paul SabatierToulouse, France

**Keywords:** long-term memory, STDP, implicit learning, temporal resolution, meaningless stimuli

## Abstract

Recent research has demonstrated that humans are able to implicitly encode and retain repeating patterns in meaningless auditory noise. Our study aimed at testing the robustness of long-term implicit recognition memory for these learned patterns. Participants performed a cyclic/non-cyclic discrimination task, during which they were presented with either 1-s cyclic noises (CNs) (the two halves of the noise were identical) or 1-s plain random noises (Ns). Among CNs and Ns presented once, *target CNs* were implicitly presented multiple times within a block, and implicit recognition of these *target CNs* was tested 4 weeks later using a similar cyclic/non-cyclic discrimination task. Furthermore, robustness of implicit recognition memory was tested by presenting participants with *looped* (shifting the origin) and *scrambled* (chopping sounds into 10− and 20-ms bits before shuffling) versions of the target CNs. We found that participants had robust implicit recognition memory for learned noise patterns after 4 weeks, right from the first presentation. Additionally, this memory was remarkably resistant to acoustic transformations, such as looping and scrambling of the sounds. Finally, implicit recognition of sounds was dependent on participant's discrimination performance during learning. Our findings suggest that meaningless temporal features as short as 10 ms can be implicitly stored in long-term auditory memory. Moreover, successful encoding and storage of such fine features may vary between participants, possibly depending on individual attention and auditory discrimination abilities.

**Significance Statement**

- Meaningless auditory patterns could be implicitly encoded and stored in long-term memory.
- Acoustic transformations of learned meaningless patterns could be implicitly recognized after 4 weeks.
- Implicit long-term memories can be formed for meaningless auditory features as short as 10 ms.
- Successful encoding and long-term implicit recognition of meaningless patterns may strongly depend on individual attention and auditory discrimination abilities.

## Introduction

Despite decades of research on the mechanisms of memory in humans, several questions regarding the spatial and temporal correlates of sensory memory remain unanswered. Sensory memory for meaningless stimuli is particularly interesting to study since memory for these stimuli in their smallest perceived units form the building blocks of sensory recognition. Further, understanding how meaningless perceptual stimuli are encoded and stored provides a template for all forms of perceptual learning during infancy. Meaningless stimuli are also interesting to use since participants cannot consciously rehearse individual exemplars and cannot hear them out of the experimental context. Evidence for our ability to detect statistical regularities in meaningless information comes from a classic study in which participants were presented with segments of auditory white noise played back to back continuously and were able to detect the recurrence of some “features” (Guttman and Julesz, 1963). Participants are also highly consistent when they tap along with the frequency of auditory noise cycling (Kaernbach, 1992), implying that they can retain meaningless information in working memory. More surprisingly, our ability to store meaningless information in long-term memory was demonstrated recently (Agus et al., 2010). Using an implicit learning paradigm, they had participants listen to Gaussian white noises while performing a cyclic/non-cyclic discrimination task. Unknown to participants, some of the cyclic sounds were presented multiple times, while others were only presented once. Participants improved at detecting cyclicity in some of the sounds presented to them multiple times and retained this knowledge after 2–3 weeks. This suggests that detection of cyclicity may have been facilitated when sounds were previously learned, i.e., improvement in the discrimination task may reflect implicit recognition of learned sound features. Moreover in the same study (Agus et al., 2010), participants listened to several exemplars of noise—which were essentially distractors—in between presentations of target noises. This suggests that memory for noise was resistant to interference effects. Interference arises from both similar noise distractors within the learning context, as well as all from environmental sounds heard during the retention period. In light of these findings, we were interested in exploring the limits and robustness of this sort of long-term memory for meaningless sounds.

A first question of interest is to determine what temporal and/or spectral features in noise are stored. Interestingly, when learned cyclic noises were played backwards during a retention test (Agus et al., 2010), detection of cyclicity was more accurate for reversed versions of learned sounds than for novel cyclic sounds, suggesting that acoustic features which are (implicitly) encoded are preserved in the reversed version of the sound. Here we further investigated how implicit memory performance varied with other acoustic transformations applied to the learned stimulus.

We also investigated the link between strength of meaningless stimuli encoding and subsequent memory performance. Turk-Brown et al. reported that brain regions such as the PPA (Parahippocampal place area, involved in scene memory) show higher activity during the encoding (first exposure) of scenes that were subsequently recalled vs. scenes that were forgotten (Turk-Browne et al., 2006) in both implicit and explicit paradigms. Performance in implicit encoding of meaningless sounds typically shows high inter-individual variability (Agus et al., 2010), and we were interested in exploring the relationship between strength of encoding and subsequent implicit recognition of these stimuli.

Putting these findings together, we hypothesized the following:

1. Long term, implicit memory for auditory noise (demonstrated as a preferential bias to detect cyclic features in learned vs. novel sounds) would be resistant to acoustic transformation, declining with increasing degree of transformation from the original learned noise.
2. This resistance to transformation would depend on how strongly the stimuli were encoded.

These claims were tested using an implicit encoding and subsequent long-term implicit recognition paradigm, as previously described (Agus et al., 2010). To test the first hypothesis, in addition to the old (learned) and novel sounds used in traditional memory retention tests, participants were presented with modified versions of the learned sounds. In some trials, the temporal origin of a learned sound was randomly shifted, changing the temporal expectancy of the learned feature(s) but preserving acoustic properties and surrounding context. In other trials, learned sounds were randomly shuffled to disrupt both temporal expectancy and surrounding context of learned features. To test the second hypothesis, implicit recognition performance on modified versions of learned sounds was considered in relation to learning performance. We predicted that implicit recognition during the retention test would vary as a function of acoustic transformation from the original learned sound. We also predicted that implicit long-term recognition would be higher in participants showing better performance during the learning session.

## Materials and methods

These hypotheses were investigated in one experiment with 2 versions. Participants were randomly assigned to one of the two versions of the experiment, which only differed in small aspects, as described in the procedure.

### Participants

A total of 37 participants between 20 and 30 years of age, with self-reported normal hearing, were screened for the experiment. Twenty-five of these participants (mean age = 24.32 years, *SD* = 3.07) were finally included. All participants were compensated for their time with gift cards pre-loaded with monetary values proportional to the extent of their participation, ranging from 10 euros (only screening) to 40 euros (completing both sessions of the experiment). They were instructed that the purpose of the experiment was to assess auditory discrimination and were naïve to the actual hypotheses of the experiment. All participants gave written informed consent in accordance to the declaration of Helsinki and the University of Toulouse and CNRS requirements for research with human participants [Protocol: CPP14-007a/2013-A01450-45].

### Stimuli

**Figure 1:**
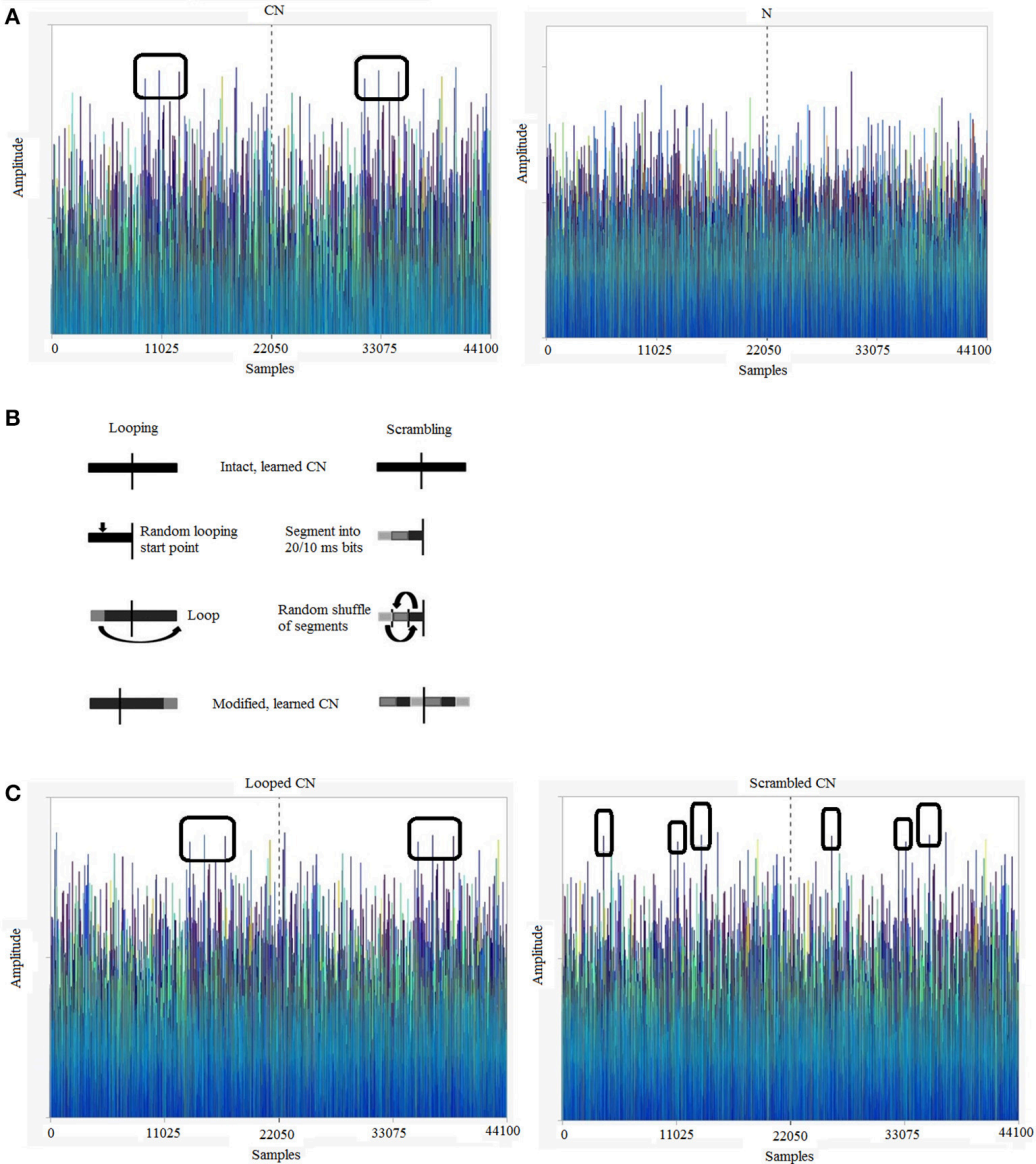
**Exemplars of 1-s Gaussian white noises (sampling frequency = 44.1 kHz) and acoustic transformations used in the experiment. (A)** Cyclic noise (CN) vs. non-cyclic noise (N): Gaussian noises typically show small amplitude variations over time. The first and second halves of a CN are identical, while an N is completely random. **(B)** Transformations used to loop and scramble the learned CNs in the testing session. For looping, a random time point was chosen in the first half of the sound and the sound portion preceding this time point was shifted to the end. For scrambling, the first half of the cyclic sound was cut into segments of 20 ms for version 1 and 10 ms for version 2, the segments were randomly shuffled and the resulting 500-ms sound was played back to back to create a scrambled CN. **(C)** Looped and Scrambled sounds: amplitude variations over time of exemplar looped and scrambled (20 ms) versions of the CN shown in **(A)**. The color scheme of **(A,C)** is graded as a function of sound amplitudes, in order to facilitate identification of repeating features.

Stimuli were programmed and generated using MATLAB R2013 (http://www.mathworks.com/). The sound stimuli were sequences of normally-distributed, 16-bit pseudo-random numbers with a zero mean, which were played at a sampling frequency of 44.1 KHz. To ensure that the sounds are different every time, we reset the seed of the pseudorandom number generator of MATLAB on every trial. We constructed Cyclic (*CN*) and Non-Cyclic (*N*) stimuli, both lasting 1 s in duration (Audio samples can be found at http://m4.ups-tlse.fr/; See Supplementary Material for Audios 1 and 2 which are exemplar CN and N, respectively). A CN was generated as a 500-ms pseudo-random segment of sound that was presented twice back to back (cycled). An N was generated as a 1000-ms pseudo-random segment. The spectrograms of such Gaussian white noises are flat, with no distinctive variations in frequency over time. Therefore, to illustrate the cyclic nature of these sounds, we plotted the actual amplitude variations over time (Figure 1A). This shows that the amplitude variations in the first and second halves are identical in a CN but not in an N. Over the experiment, participants were presented with 4 variations of the CNs (explained below) while all the Ns were uniquely generated and heard only once. The generation and exemplar amplitude variations in modified CNs are illustrated in Figures 1B,C.

#### Target CN

This was a uniquely generated CN that was presented several times to the participant over the learning and testing sessions of the experiment.

#### CN

This was a uniquely generated CN that was heard only once throughout the experiment.

#### Looped target CN

This was a modified version of a target CN. For looping a target CN, a random time point was chosen from its first half, the sequence was cut at this point and the preceding segment was pasted at the end.

#### Scrambled target CN

A modified version of a target CN was created by segmenting the first half (500 ms) into several bins of equal size, which were randomly shuffled and then played back to back to create a CN.

It is important to note that each presentation of a looped or scrambled CN was different to prevent learning of one exemplar of the looped/scrambled version of the target CN throughout the session. Looped and scrambled CNs were presented to the participants only during the testing session.

**Figure 2:**
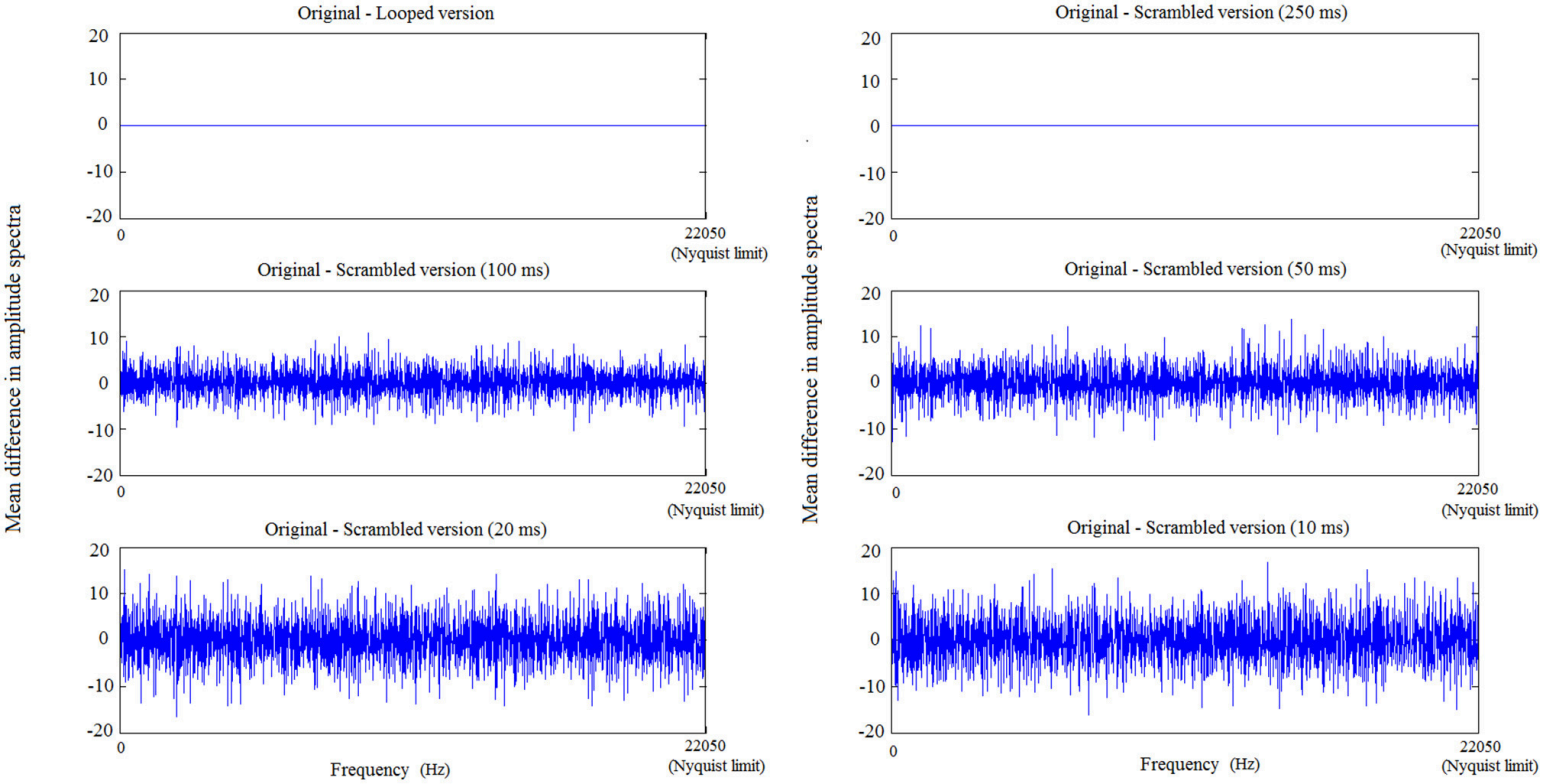
**Changes in frequency features—in low, mid and high frequency bands—of a CN due to looping and scrambling with increasing bin sizes**. The maximal frequency on the X axis corresponds to the Nyquist frequency (22,050 Hz) and the spectrum amplitude *difference* between original and looped/scrambled versions of a CN is plotted on the Y axis. With decreasing bin size, the difference between the resulting scrambled sound and the original sound increases, leading to greater difference in amplitude spectrum from the original, across all the frequency bands.

To further understand how scrambling and looping affect the acoustic properties of a CN, we calculated Fourier transforms of an exemplar CN and its variants. Variants were created similar to the looped and scrambled sounds. Bin sizes of 250, 100, 50, 20, and 10 ms were used to create 5 distinct scrambled versions of the exemplar CN. The difference in amplitude between spectra of these variants and spectrum of the original CN is plotted in Figure 2, with frequency bins (10 samples/bin) ranging from lower bands to higher bands on the X axis. While looping and 250-ms scrambling does not change the amplitude spectrum at any frequency, scrambling using 100-ms or smaller bins affects the amplitude spectrum at all frequencies.

### Task

All participants performed 2 sessions of a forced-choice discrimination task, 4 weeks apart. Each trial started with participants hearing a Gaussian noise of 1 s, after which they had to discriminate the sound as cyclic/non-cyclic. Participants did not receive any feedback about their performance. All trials were presented in a randomized order. After session 1, each participant's performance was analyzed (as explained in the analysis section) and learned target CNs were selected for the session 2. Participants on average took about an hour to complete each experimental session, not including training. The training session took on average 15 min. We provided participants with scheduled breaks between blocks and participants were informed that they could also pause within a block to take breaks as necessary.

### Procedure

Both experimental sessions included 10 blocks of 80 trials each. The first session included a training part followed by an implicit learning part. The second session was the testing part.

Each participant was assigned to perform one of 2 versions of the study. Differences between both versions are explained below.

#### Session 1: training

Before starting the learning part of the experiment, all participants underwent a training session, during which they listened to CNs and Ns of varying durations. This training was intended to habituate participants to detect cyclic patterns in random noise. Each training stage was repeated with new sounds until participant reached performance criterion. To explain the difference between cyclic and non-cyclic sounds, participants first listened to samples of 5-s cyclic sounds constructed as 10 repeats of a 500 ms random noise segment and 5 s non-cyclic random noise sounds until they could confidently differentiate between the two types of sounds verbally. Participants started the training phase by listening to 5 CNs (5 s, 10 repeats of 500 ms segment) and 5 Ns (5 s), in random order (training stage 1). After each sound was presented, participants had to indicate via a keyboard button press if the sound was cyclic or not. They were then given feedback about their response. Once they had correctly identified all CNs they moved to the stage 2, during which they were presented with 20 CNs (2 s, 4 repeats of a 500 ms segment) and 20 Ns (2 s). Once participants achieved a global accuracy of 80% of correct responses for the CNs they moved to the stage 3 and were presented with 20 CNs (1.5 s, 3 repeats of a 500 ms segment) and 20 Ns (1.5 s) until they achieved a global accuracy of 70% of the CNs. At any stage of the training, participants who did not reach criterion ended their participation in the study. The training was identical for both versions of the experiment.

#### Session 1: learning

Participants performed 10 blocks of the forced-choice discrimination task (as described earlier) immediately after training. In each block, participants were presented with 40 *Ns*, 20 *CNs*, and 20 repeats of a unique *target CN*.

##### Version 1

Each participant was randomly assigned to 1 of 2 possible sets of 10 target CNs. This was aimed at testing the existence of any systematic biases to detect cyclicity in some target CNs over others.

##### Version 2

All participants heard the same set of 10 target CNs (set 2 of version 1).

#### Session 2: testing

As far as the participant was concerned, this session consisted in an identical forced-choice discrimination task, similar to the one performed in the learning session. However, the stimuli used were different: for each participant we created a list of the “learned” target CNs (discrimination performance of at least 80%, i.e., a sound which participants correctly discriminated as cyclic at least 16 out of the 20 times they heard it), which was further used to create looped and scrambled CNs. In addition to Ns, CNs, and Target CNs, participants were presented with looped CNs during 5 blocks and with scrambled CNs during the other 5 blocks (block order was randomized). Each block included 40 *Ns*, 10 *CNs*, 10 *target CNs* (chosen randomly on each trial from a list of *learned target CNs* for each participant) and 20 *modified (looped/scrambled) target CNs*. The scrambled sounds that participants heard were different based on the version they had been assigned to, as explained below.

##### Version 1

Participants assigned to version 1 of the experiment were presented with learned target CNs scrambled using 20-ms time bins. That is, the first half of a learned target CN was cut into 25 bins of 20 ms (882 samples in each bin) before shuffling to create a scrambled target CN.

##### Version 2

Participants assigned to version 2 of the experiment were presented with learned target CNs scrambled into 10-ms time bins. That is, the first half of a learned target CN was cut into 50 bins of 10 ms (441 samples in each bin) before shuffling to create a scrambled target CN.

Finally, we were interested in analyzing how two parameters—sleep and sound imagery—might influence learning and memory in our paradigm. Numerous studies have shown the influence of quality of sleep (review, Walker and Stickgold, 2014) in learning and memory for different types of stimuli. To assess quality of sleep, participants maintained a sleep diary, similar to those used previously (Mary et al., 2013), during the 4 weeks between learning and testing sessions. During the testing session, Participants also filled out St. Mary's sleep questionnaire (Ellis et al., 1981) regarding their last night's sleep quality. Lastly, to assess the influence of sound imagery on the ability to do the discrimination task, participants also filled out a sound imagery questionnaire (Willander and Baraldi, 2010).

### Analysis

Analysis was done using MATLAB and statistical tests were performed using JMP (Version 12. SAS Institute Inc., Cary, NC, 1989-2007).

#### Learning session analysis

The proportion of hits and false alarms in each block was calculated, for all participants. The correct identification of a CN (CNs and target CNs) was considered a hit and the incorrect identification of an N as a cyclic noise was considered a false alarm. All target CNs that were correctly identified in at least 80% of the trials were considered *learned target CNs*. The list of *learned target CNs* was subsequently used to create the testing session stimuli for each participant.

Moreover, we investigated any systematic biases in detecting cyclicity in some target CNs over others. For each target CN presented in the learning session, the proportion of participants who actually learned the sound was determined. This was done for each set of target CNs over the two versions of the experiment.

Individual discrimination performance was computed over the 10 blocks, using the principles of signal detection theory. We calculated individual *a'*, a non-parametric measure of participants' sensitivity to differences between signal (target) and noise (distractor), i.e., cyclic vs. non-cyclic stimuli (Pollack and Norman, 1964; Stanislaw and Todorov, 1999). While sensitivity is traditionally evaluated using d', an assumption for using d' is that signal and noise distributions have equal standard deviations. In our experiment, since the signal trials include different subtypes of trials (CNs or target CNs) but not the noise trials (Ns), a' is a better estimate for sensitivity than d'. A' was calculated using the formula provided by Stanislaw and Todorov:

(1) $$\begin{array}{l} \text{A}\prime =0.5\;+\;\left[\text{sign}(\text{H}-\text{F}){\left(\text{H}-\text{F}\right)}^{2}+\frac{\text{|H}-\text{F|}}{4*\text{max}(\text{H},\text{F})-4*\text{HF}}\right]\\ \text{ Sign}(\text{H}-\text{F})\;=\;\text{+}1\;\text{if}\;(\text{H}-\text{F})>0\;\text{and}\;-1\;\text{if}\;(\text{H}-\text{F})<0 \end{array}$$

Where H = proportion of Hits for the signal trials and F = proportion of False Alarms for distractor trials.

Any participant with a' < 0.5 was excluded from the analysis (and from subsequent participation in the testing session) since this implied that this participant's performance was at chance level.

#### Testing session analysis

The proportion of hits (correct identification of *learned target, looped, scrambled*, and *novel CNs* as cyclic) and false alarms (incorrect identification of *Ns* as cyclic) was calculated for each individual. The discrimination rate for CNs was determined individually as the number of times (out of 20 presentations within a block) a CN was correctly discriminated.

To differentiate between participants who were merely good at detecting noise cyclicity from those demonstrating a preferential bias toward previously learned cyclic sounds, i.e., an implicit memory effect, we compared discrimination rates for *learned target* and *novel CN* trials in each individual. A participant who had truly learned a *target CN* would more accurately detect cyclicity for this noise over a *novel CN*. To ensure that any observed preferential bias to discriminate learned target CNs was not due to within-session rapid learning, the discrimination rate for learned target CNs was also analyzed as a function of time.

Moreover, to investigate the relationship between how well a sound was learned, quantified as discrimination performance in the learning session, and subsequent memory resistance to acoustic transformations, we compared a' during learning (a'^learning^) with discrimination rate for *intact learned, looped*, and *scrambled target CNs* in the testing session. A high a'^learning^ would mean participants accurately detect cyclicity during the learning session.

Lastly, scores from the questionnaires on sleep quality and sound imagery were correlated with a' values across our group of participants.

## Results

**Figure 3:**
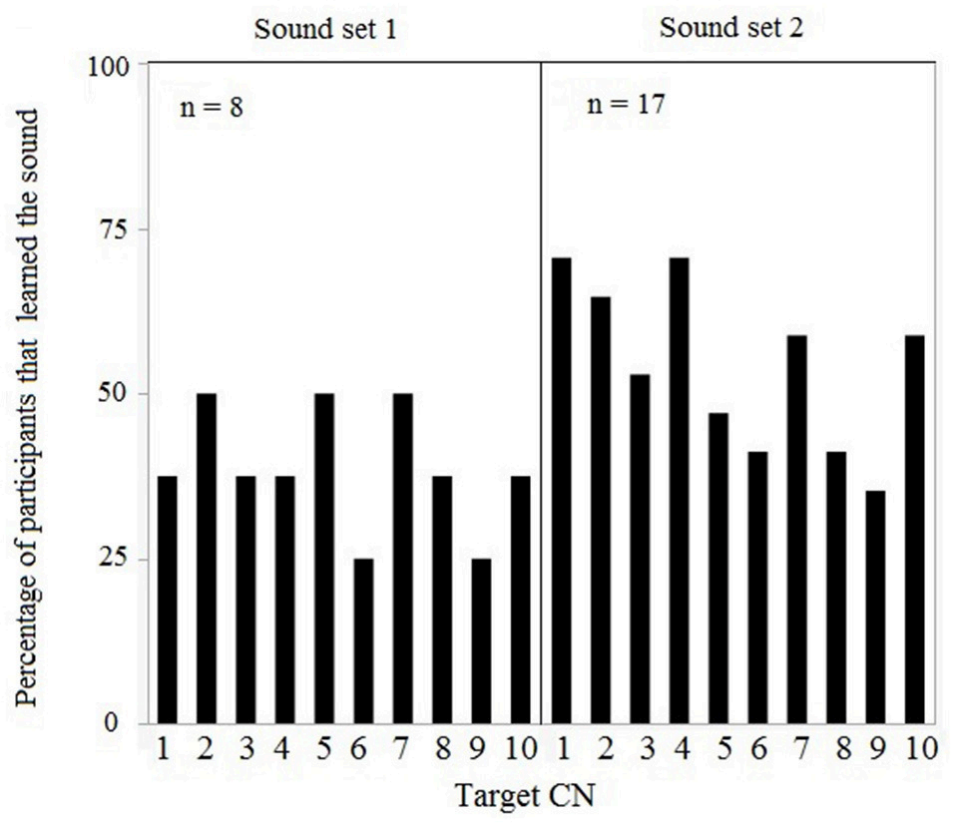
**Learning session results for both sets of 10 target CNs: Each target CN was learned by a variable percentage of participants, i.e., there were no target CNs that were systematically learned by all participants**.

Based on individual performances in the training and learning session [inclusion criteria: a' > 0.5], data from 16 (of 26) participants in version 1 and data from 9 (of 11) participants in version 2 were included in the analyses. Since the learning session followed an identical procedure and resulted in equivalent discrimination sensitivity (a' ^learning^) in both versions [*F*_(1, 25)_ = 0.4287, *p* = 0.52], data from the first session for all 25 participants were pooled. Data from the training session for both versions is summarized in Supplementary Figure 1. Individual a' values ranged between 0.53 and 0.97 (mean a' = 0.73, *SD* = 0.71). The number of sounds learned by participants within each set of target CNs was computed, showing no preferential bias for some sounds over others in set 1 [*F*_(9, 80)_ = 0.26, *p* = 0.98] and set 2 [*F*_(9, 170)_ = 1.09, *p* = 0.37] (Figure 3). Moreover, the proportion of participants who learned the target CNs did not differ between both sets [*F*_(9, 20)_ = 0.24, *p* = 0.98] (Figure 3).

**Figure 4:**
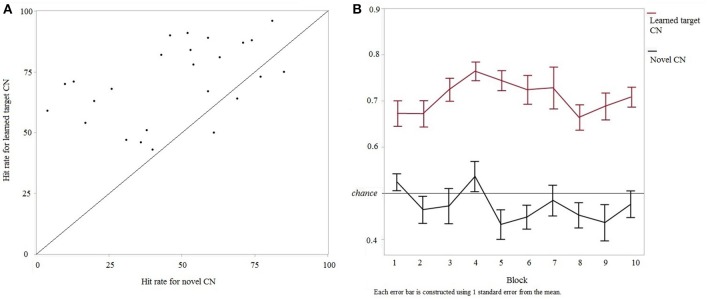
**Discrimination performance for intact learned target CNs vs. novel CNs in the testing session. (A)** Relationship between discrimination rates of learned target and novel CNs in each participant. Participants above the diagonal show higher rates for learned vs. novel CNs, suggesting that memory facilitated the discrimination task. **(B)** Discrimination rates of learned target and novel CNs over time (10 blocks).

Participants performed the testing session a month after the learning session (mean interval 30.96 ± 4.2 days, range 23–41 days). In the testing session, discrimination sensitivity (a' ^testing^) again did not vary between versions 1 and 2 of the experiment [*F*_(1, 25)_ = 0.0057, *p* = 0.94] and therefore these data were pooled (*n* = 25). Detection of cyclicity in novel CNs did not change across the two sessions [Mean difference = 0.58, *SE* = 3.8, *t*_(25)_ = 0.15, *p* = 0.8803] indicating that participants' performance in the task was similar over the 4 weeks. Furthermore, the number of times a participant performed each training stage during the learning session had no effect on the discrimination performance during the testing session (a' testing): training stage 1 [*F*_(1, 25)_ = 0.09, p = 0.75], training stage 2 [*F*_(1, 25)_ = 1.98, *p* = 0.17] and training stage 3 [*F*_(1, 25)_ = 0.03, p = 0.86]. Within the testing session, discrimination rates were significantly higher for learned target CNs compared to novel CNs [*F*_(1, 25)_ = 7.03, *p* < 0.014] (Figure 4A). This suggests that participants had memory for the CNs previously learned in the first session. Furthermore, to ensure that this higher discrimination rate for learned target CNs did not result from learning of features throughout the testing session (as opposed to long-term memory for features from the first session), the evolution of discrimination rates for learned vs. new CNs was analyzed over time. A two-way repeated-measures ANOVA on discrimination rates was computed, testing main effects and interaction of within-subjects factors “trial type” (2 levels, “learned target CN” and “novel CN”) and “block” (10 levels). Trial type was the only significant predictor of performance [*F*_(1, 200)_ = 313.696, *p* < 0.0001] irrespective of block [*F*_(9, 200)_ = 1.57, *p* = 0.127]. The effect of trial type was equivalent across blocks [*F*_(9, 200)_ = 1.06, *p* = 0.394]. These results were confirmed by the absence of correlation between hit rate for learned and novel CNs over the 10 blocks [*F*_(1, 100)_ = 0.14, *p* = 0.71; *R*^2^ = 0.001, slope = −0.03, intercept = 0.7, *p* = 0.71]. These results are shown in Figure 4B. Progression of the other trial types, i.e. looped and scrambled learned CNs, are summarized in Supplementary Figure 2.

**Figure 5:**
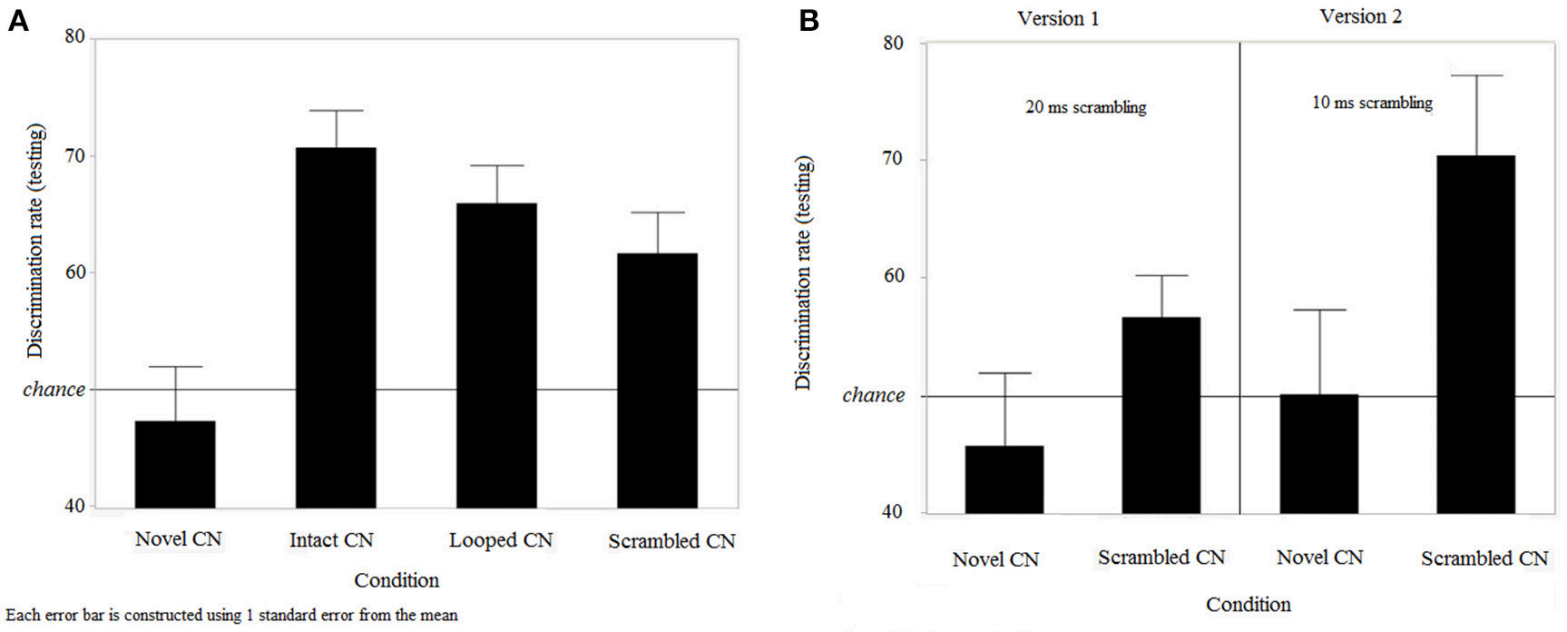
**Discrimination performance during the testing session. (A)** Performance for intact, looped, scrambled learned target CNs and novel CNs (*n* = 25) and **(B)** Discrimination performance for scrambled trials with 20 and 10 ms bin sizes (*n* = 16 in version 1 and *n* = 9 in version 2).

Since participants had long-term memory for learned target CNs, we further analyzed discrimination rates for all types of CNs in the testing session. Since scrambled target CNs were different in versions 1 and 2 of the experiment, the effect of version on discrimination rates was specifically tested. A two-way ANOVA was conducted, using within-subjects factor of “trial type” (4 levels, “intact target CN,” “looped target CN,” “scrambled target CN,” and “novel CN”) and between-subjects factor of “version” (2 levels). A significant effect of trial type on discrimination rates was found [*F*_(3, 100)_ = 23.73, *p* < 0.0001]. There was no effect of version on discrimination rates [*F*_(1, 100)_ = 2.29, *p* = 0.1432], and no interaction between both factors [*F*_(3, 100)_ = 0.99, *p* = 0.4]. Since there was no evidence for any effect of version or interaction, data were pooled across both versions (Figure 5A) and differences between trial types were further examined. Tukey's Honestly Significant Difference (Tukey's HSD) tests showed that discrimination rates for novel CNs were lower than discrimination rates for all other CNs; that is, detection in intact target [effect size (mean_(*i*)_-mean_(*j*)_) = 24.6, CI_95%_ = (16.5, 32.7), *p* = 0.0001], looped [effect size = 19.23, CI_95%_ = (11.1, 27.3), *p* = 0.0032] and scrambled [effect size = 15.67, CI_95%_ = (7.6, 23.7), *p* = 0.0362] CNs were all significantly higher than novel CNs. Discrimination rates for intact target and looped trials were equivalent [*p* = 0.8004], and so were the discrimination rates for looped and scrambled trials [*p* = 0.8464]. However, discrimination rates for learned intact trials were higher than for scrambled trials [effect size = 8.93, *SE* = 3.07, *p* = 0.3164]. We were also interested in any performance difference for the scrambled trials between versions 1 and 2, since the bin sizes were different in the two versions. As shown from the two-way ANOVA, discrimination rates were not impacted by the version of the experiment, indicating that scrambling learned CNs with bin sizes of 10 or 20 ms resulted in equivalent performance. For information, we report in Figure 5B results for scrambled CNs, for the 2 versions separately.

**Figure 6:**
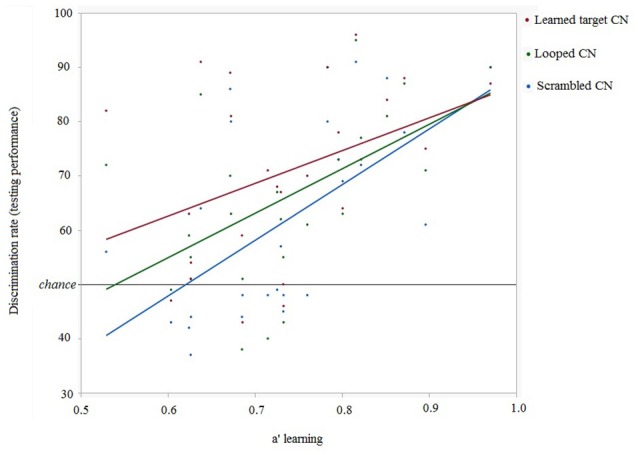
**Relationship between discrimination rates of CNs in the testing session and learning efficiency (represented as a') for all participants (*n* = 25)**.

Individual discrimination performances in the testing phase as a function of discrimination efficiencies (quantified as a') during the learning phase for learned CNs and modified forms of the learned CNs were examined. Linear regression between hit rate_(*testing*)_ and a'_(*learning*)_ showed that a' during learning did predict later detection of cyclicity in looped [*R*^2^ = 0.272, slope = 81.95, intercept = 5.71, *p* = 0.0075] and scrambled CNs [*R*^2^ = 0.366, slope = 102.59, intercept = −13.73, *p* = 0.0013]. The correlation between a' during learning and discrimination rate for intact learned CNs [*R*^2^ = 0.153, slope = 60.36, intercept = 26.32, *p* = 0.053] was just below significance. These results show that a' during learning was a significant predictor of further accuracy to discriminate modified versions of learned CNs 4 weeks later. This is shown in Figure 6.

Lastly, the relationship between discrimination performance in the learning and testing sessions and parameters quantified using sleep and sound imagery questionnaires was investigated. The results are summarized in Supplementary Figure 3. There was no correlation between discrimination ability in the testing phase and self-reported sleep quantity [*R*^2^ = 0.02, slope = 0.007, intercept = 0.7, *p* = 0.55] or quality of sleep [*R*^2^ = 0.004, slope = −0.002, intercept = 0.79, *p* = 0.76] the night before testing. There was no correlation between discrimination rates and alertness the day of testing [*R*^2^ = 0.05, slope = 0.02, intercept = 0.69, *p* = 0.29]. We also found no link between sound imagery scores, as assessed by St Mary's questionnaire, and discrimination in the learning [*R*^2^ = 0.07, slope = −0.03, intercept = 0.85, *p* = 0.32] and testing [*R*^2^ = 0.09, slope = −0.04, intercept = 0.89, *p* = 0.23] sessions.

## Discussion

The present results confirm our hypothesis that features in meaningless sounds can be learned and retained over several weeks. The results also demonstrate the *robustness* of this memory to acoustic transformations: despite a decrease in the preferential bias to detect learned features with increasing degree of transformation from the original, participants were more accurate to detect cyclicity in highly degraded versions of learned sounds in comparison to novel cyclic sounds. The *quality of learning* was a predictor of this memory to survive acoustic transformation. While models of sleep and memory predict that stored features are subject to opposing factors that selectively strengthen (reactivations during sleep) (Rudoy et al., 2009) and weaken (internal pruning based on probability estimations of re-occurrence) (Kim et al., 2014) the memory trace, sleep parameters quantified by self-report measures did not correlate with discrimination performance; more objective measures of sleep are necessary to understand the role of sleep in memory for Gaussian sounds.

Regarding robustness of memory to acoustic transformations, we found that participants had equivalent implicit recognition memory for intact and looped (onset-shifted) versions of a learned sound, clearly demonstrating that feature learning was not restricted to sound onset. Instead, learned acoustic features that facilitate implicit recognition may be scattered throughout the sound. We also surprisingly observed long-term implicit recognition of scrambled versions of learned sounds, where only small bin sizes of 10 and 20 ms were preserved and temporal context of learned features was lost. Although the limit of memory capacity has been discussed since Miller proposed the concept (Miller, 1956), few studies have investigated the capacity limits of implicitly-encoded purely sensory memory. The upper capacity limit in working memory for Gaussian noises was found to be around 100 ms for individual spectro-temporal features, while the lower resolution limit was unclear (Kaernbach, 1993, 2004). Our results demonstrate that this lower resolution limit could be as short as 10 or 20 ms. Since each presentation of a scrambled sound was randomly generated, new features greater than 10 ms in length could not be learned throughout testing. As shown in our analysis, scrambling modifies the spectral features of original sounds as a function of bin size, with these modifications staying nearly uniform across higher and lower frequency bands. This observation renders the coding of sound frequency features an unlikely mechanism to explain long-term implicit recognition. An alternative explanation would be that participants were able to store temporal features shorter than 10 ms. Interestingly, participants who accurately discriminated cyclic and non-cyclic sounds during the learning session also had higher implicit recognition memory for looped and scrambled versions of learned CNs, suggesting that the size of a stored feature is inversely proportional to encoding efficiency. Our data also demonstrate that these learned features vary between participants, and that no single feature could be learned by all participants. The phenomenon of stochastic resonance, where *optimal*^1^ noise can enhance the periodicity of a weak signal causing the signal to rise above the threshold for detection (Wiesenfeld and Moss, 1995), puts this finding into perspective. This phenomenon was demonstrated in the somatosensory system (in anesthetized cat): periodic tactile stimuli, which had been optimally enhanced through addition of noise, evoked field potentials (Manjarrez et al., 2003). Similarly, we speculate that specific acoustic features (weak signal) of a Gaussian sound may be preferentially enhanced when added with baseline neural activity (optimal noise) for a given individual, resulting in different features being encoded by different participants. Further support for this hypothesized mechanism of learning features in meaningless stimuli comes from a recent study where the authors asked participants to perform a similar discrimination task in an MEG scanner (Luo et al., 2013). The authors found that the phase of auditory cortical neural responses change and track learning of target CNs in the theta (3–8 Hz) range. They also demonstrate that different learned target CNs induce diverse phase pattern responses. These results suggest that as features in target CNs are learned, phase-mediated temporal encoding specific to the learned feature occurs in the auditory cortex. Since white noise (which doesn't contain acoustic features or edges) does not reset the phase of ongoing oscillations (Luo and Poeppel, 2012), stochastic resonance could contribute to feature detection. These ideas raise interesting hypotheses for future testing.

Recently, the neural correlates of memory for meaningless sounds were investigated in a study where participants were presented with 200- or 500-ms patterns that repeated every 500 ms (Andrillon et al., 2015). These patterns were embedded randomly in 8 min of continuous noise while participants detected changes in amplitude modulations. Fully developed evoked potentials were observed within 5 presentations of a repeating pattern suggesting that even in the absence of a task the brain can learn patterns within a few exposures. These evoked potentials (in terms of amplitude, coherence and spectral power) correlated with auditory discrimination performance. Consistently, our results suggest that individual factors influencing neural activity, such as attention, impact the encoding of acoustic features.

Our results are not in line with traditional models of sensory memory (for a review of experiments that led to these models, refer to Cowan, 1988). Sensory memory, such as memory for Gaussian noises, has been proposed to be characterized by 4 features—(a) it forms independently of attention, (b) it is modality specific, (c) it has fine resolution, and (d) it has a short retention time, thereby distinguishing it from categorical memory which is held in long term memory. Cumulative evidence against this model of sensory memory was discussed by Winkler and Cowan (2005) in light of results from auditory memory reactivation studies. Results from several studies using the mismatch negativity paradigm suggest the existence of longer lasting memory for acoustic regularities that are associated with “anchoring” features of a stimulus. What these anchoring features might be, or how these regularities might be stored are not clear and the authors argued for a need for better models to explain sensory memory.

As argued by Winkler and Cowan, our data suggest a need for a better model to explain mechanisms of auditory sensory memory. Data from our scrambling condition show that there is long-term memory for purely sensory features. Additionally, a' during learning influenced the robustness of implicit recognition memory. Fluctuations in the attentional network as well as bottom-up, feature-based attention invoked by the stimuli may have affected participants' accuracy to differentiate cyclic and non-cyclic sounds during learning and memory formation, therefore challenging the first claim. This is further supported by individual differences in encoding a given noise feature. The fact that participants retained their preferential bias over several weeks also challenges the 4th claim (short retention time). Rather, our results are more in line with predictions from the emergent memory account (EMA) (Graham et al., 2010). According to this model, the boundary between sensory perception and memory is not clearly defined and memory emerges as a result of hierarchical organization of perceptual representations that are distributed throughout the brain. Thus, this model predicts that sensory memories can rapidly form as a function of attention and number of presentations. Our results are compatible with these predictions and memory for Gaussian noise is likely the result of detecting repeating spike patterns. Attention modulates the sensory representations of these features while number of presentations influences the probability of feature detection. The models discussed by Winkler and Cowan (Winkler and Cowan, 2005) and the EMA (Graham et al., 2010) are quite different in their explanations of how sensory memory works since the EMA model is a model of all types of memory. Although our data challenge traditional models of sensory memory, further experiments specifically comparing observed (experimental data) vs. predicted (from models) features of memory need to be conducted to understand the mechanisms of sensory memory in light of these varied models of memory.

Further support for the claim that short acoustic features are rapidly (Andrillon et al., 2015) and robustly (our results) stored, comes from Spike time dependent plasticity (STDP) models that demonstrate how neurons can learn repeating spatiotemporal patterns in noise (Masquelier et al., 2008, 2009). The model used random Poisson activity in 2000 afferents with variable instantaneous firing rates at baseline. Continuous spike trains were then fed to this neuron detecting spike coincidences, and an arbitrary pattern was randomly repeated in the input stream. The target pattern consisted of 50 ms of spiking activity copy-pasted at random intervals in a subset of the afferents and the neuron specialized to respond with 100% selectivity (0 false alarms) to the target pattern within few tens of presentations, demonstrating fast unsupervised learning. After learning of the target pattern, a small fraction of the synapses had become selective to the pattern (383/2000 afferents) and the rest were completely silent. Interestingly, while chance determined which part of the 50 ms target pattern the neuron learned, the first spike was observed as early as 4 ms after target pattern onset, suggesting that really small features of repeating patterns were detected. The Gaussian sounds used in our study would induce firing patterns in the auditory nerve similar to spike patterns observed in the afferents of this model (Masquelier et al., 2008), Furthermore, cortical neurons with firing rates of 25 Hz or lower have been shown to function as coincidence detectors (König et al., 1996). While Konig and colleagues studied cortical neurons, according to STDP any low firing rate, coincidence detecting neuron, either cortical or sub-cortical, could learn meaningless repeating patterns. Since participants had memory for 10- and 20-ms scrambled versions of learned sounds, a few neurons that fire at/below 25 Hz acting as coincidence detectors probably specialized to respond to extremely short features in learned target CNs.

To conclude, using the frozen noise paradigm in an implicit learning protocol, we showed that participants had robust implicit recognition memory for short temporal features of meaningless sounds. The robustness of this memory may depend on individual encoding strength. Further research is required to understand the neural mechanisms underlying memory for meaningless stimuli.

## Author contributions

JV took part in experimental design, data collection, data analysis and writing the paper. FR took part in experimental design, data analysis and writing the paper. NB took part in experimental design and supplied research tools. ST took part in experimental design, writing the paper and supplied research tools and infrastructure.

### Conflict of interest statement

The authors declare that the research was conducted in the absence of any commercial or financial relationships that could be construed as a potential conflict of interest.

## Footnotes

^1^The noise has to be optimal since too much noise can mask the weak signal completely and too little noise does not lead to a correlation between the weak signal and detection of events.

## Supplementary material

Supplementary Figure 1 **Performance during the training session, representing number of runs to reach criterion before moving onto the next stage**. Participants who did not reach criterion within 5 runs of any stage discontinued the experiment.

Supplementary Figure 2 **Progression of hit rates for looped and scrambled CNs across the testing session**. During testing, looped CNs were presented during half of the blocks, i.e., 5 blocks, and the other half included scrambled CNs. As for learned target CN (Figure 4B), discrimination rate for looped and scrambled trials are above chance from the first block.

Supplementary Figure 3 **Correlations between sleep quality, sound imagery and discrimination rates of CNs (measured as a')**. Clockwise from the top-left: **(A)** Correlation between sound imagery (measured using the French version of Willander and Baraldi, 2010) to learning and testing performance. **(B)** Correlation between self-reported sleep quality (measured from a subset of questions from the St. Mary's sleep questionnaire) and testing performance. **(C)** Positive correlation between self-reported alertness the day of the testing session (measured from another subset of questions from the St. Mary's sleep questionnaire) and testing performance. **(D)** Positive correlation between self-reported sleep quantity (measured from a third subset of questions from the St. Mary's sleep questionnaire) and testing performance. Overall, none of the parameters we measured significantly influenced discrimination performance.
