# Supplementary figures and images for: Long Term Memory for Noise: Evidence of Robust Encoding of Very Short Temporal Acoustic Patterns
Source: Front Neurosci. 2016 Nov 24;10:490. doi: 10.3389/fnins.2016.00490 (PMC5121232; doi:10.3389/fnins.2016.00490)

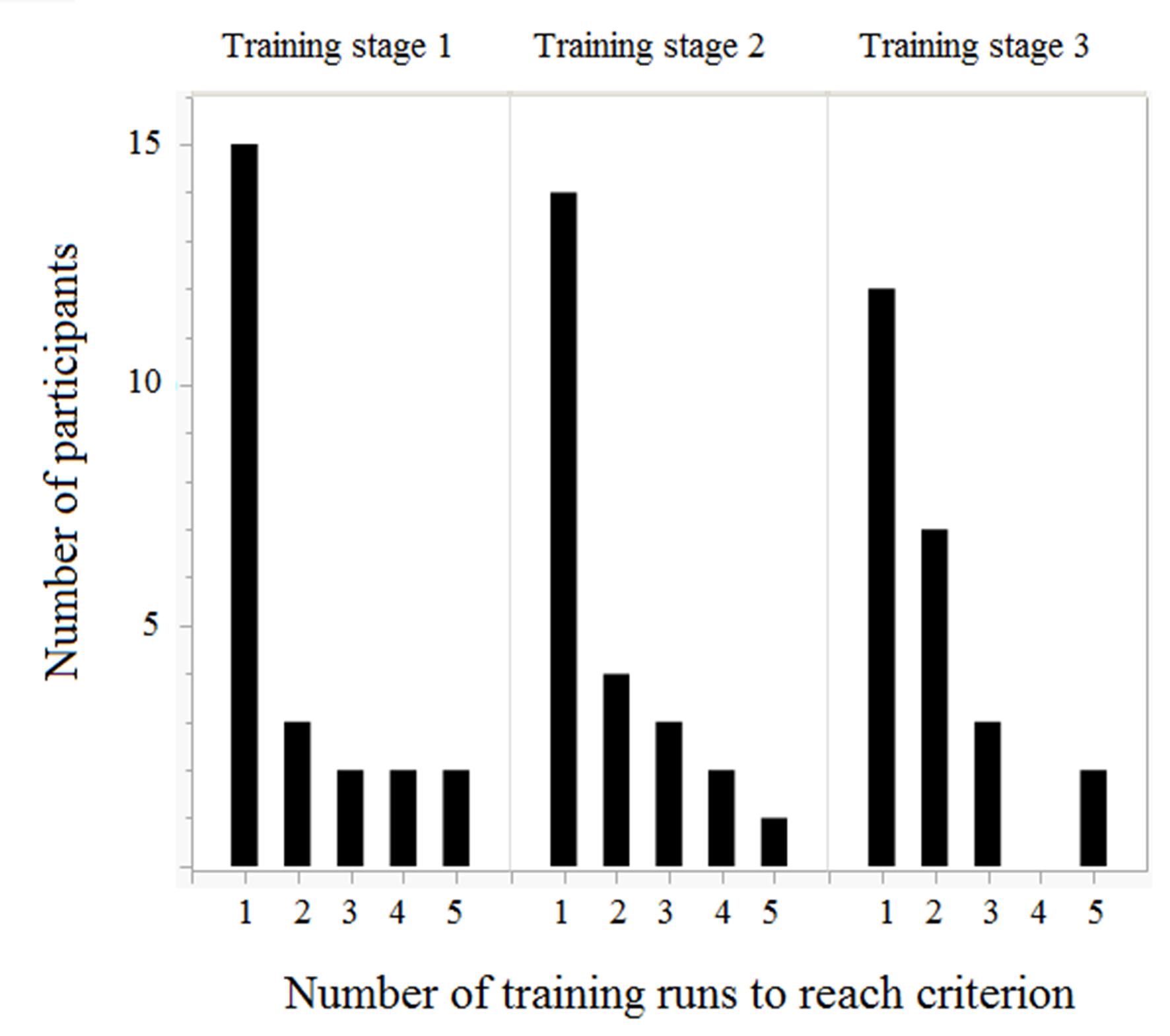

Supplement: Supplementary Figure 1 — Performance during the training session, representing number of runs to reach criterion before moving onto the next stage. Participants who did not reach criterion within 5 runs of any stage discontinued the experiment. [file Image1.TIF]

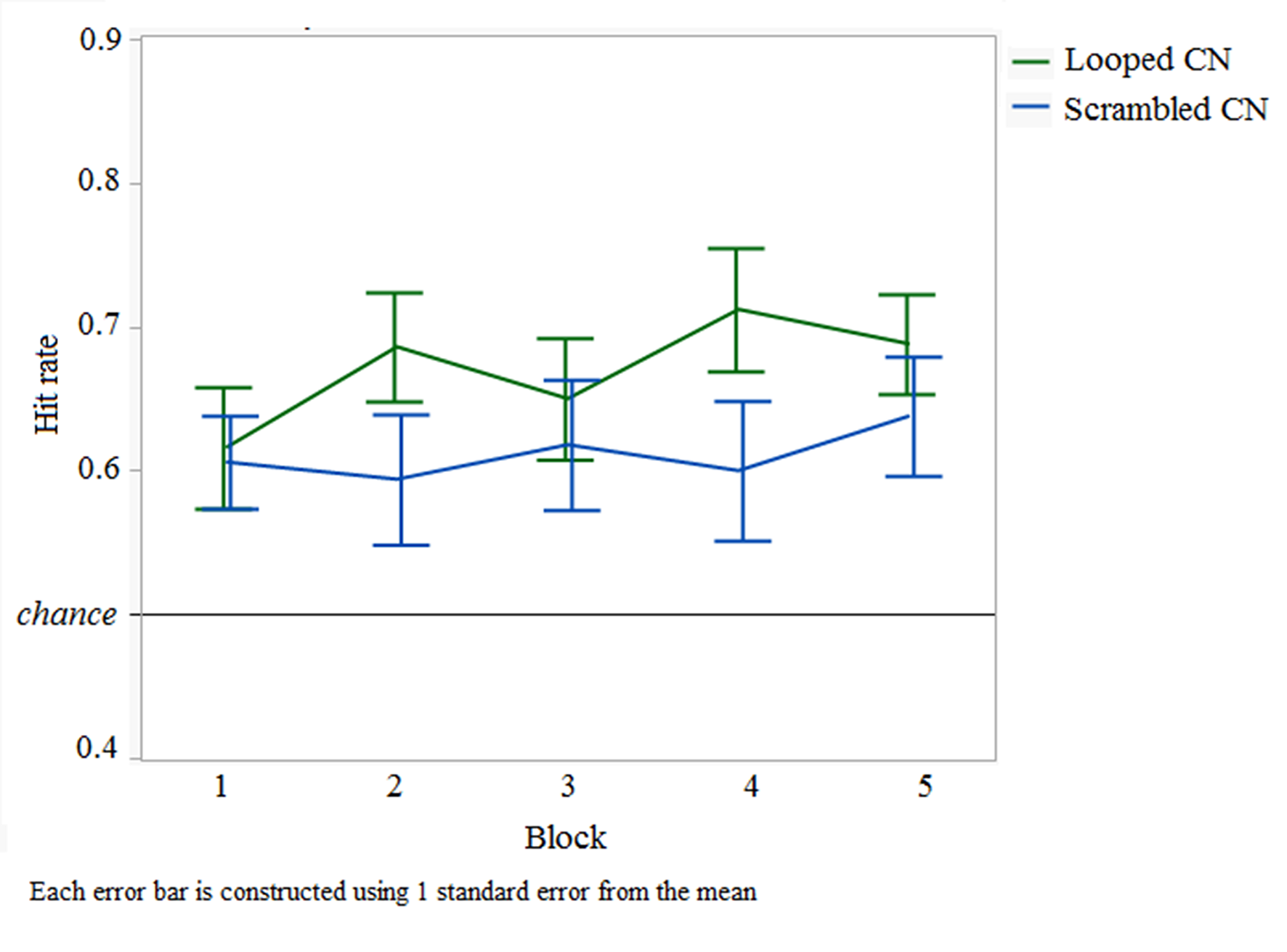

Supplement: Supplementary Figure 2 — Progression of hit rates for looped and scrambled CNs across the testing session. During testing, looped CNs were presented during half of the blocks, i.e., 5 blocks, and the other half included scrambled CNs. As for learned target CN (Figure 4B), discrimination rate for looped and scrambled trials are above chance from the first block. [file Image2.TIF]

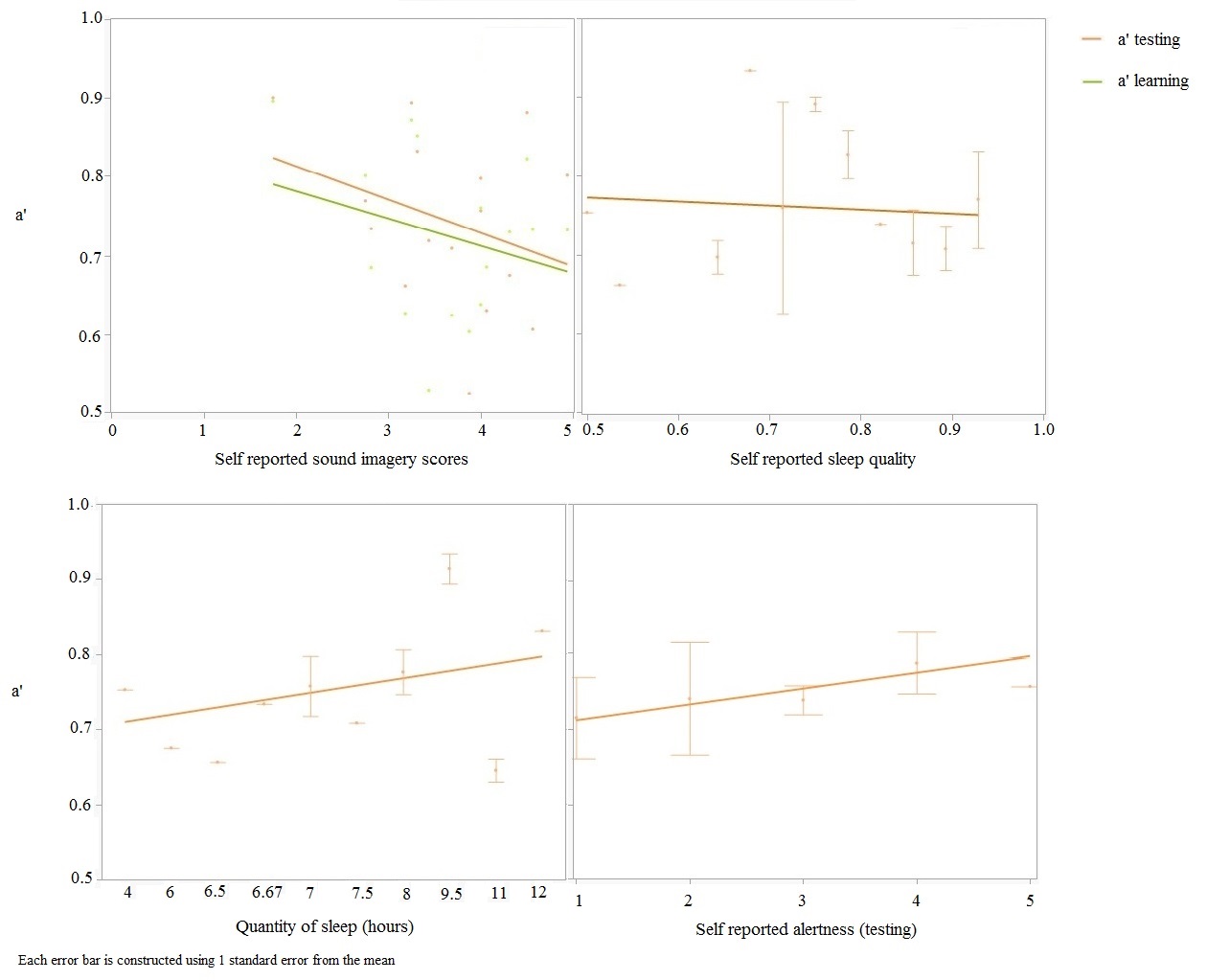

Supplement: Supplementary Figure 3 — Correlations between sleep quality, sound imagery and discrimination rates of CNs (measured as a'). Clockwise from the top-left: (A) Correlation between sound imagery (measured using the French version of Willander and Baraldi, 2010) to learning and testing performance. (B) Correlation between self-reported sleep quality (measured from a subset of questions from the St. Mary's sleep questionnaire) and testing performance. (C) Positive correlation between self-reported alertness the day of the testing session (measured from another subset of questions from the St. Mary's sleep questionnaire) and testing performance. (D) Positive correlation between self-reported sleep quantity (measured from a third subset of questions from the St. Mary's sleep questionnaire) and testing performance. Overall, none of the parameters we measured significantly influenced discrimination performance. [file Image3.JPEG]
